# Adverse reproductive effects of S100A9 on bovine sperm and early embryonic development in vitro

**DOI:** 10.1371/journal.pone.0227885

**Published:** 2020-01-16

**Authors:** Natsumi Funeshima, Nao Tanikawa, Hikari Yaginuma, Hiroyuki Watanabe, Hisataka Iwata, Takehito Kuwayama, Seizo Hamano, Koumei Shirasuna

**Affiliations:** 1 Department of Animal Science, Tokyo University of Agriculture, Atsugi, Kanagawa, Japan; 2 Animal Bio-Technology Center, Livestock Improvement Association of Japan Inc., Tokyo, Japan; 3 Department of Life and Food Sciences, Obihiro University of Agriculture and Veterinary Medicine, Obihiro, Hokkaido, Japan; 4 Maebashi Institute of Animal Science, Livestock Improvement Association of Japan Inc., Gunma, Japan; School of Sciences and Languages, Sao Paulo State University (UNESP), BRAZIL

## Abstract

The phenomenon of aging arises from multiple, complex interactions causing dysfunction in cells and organs. In particular, fertility drastically decreases with age. Previously, we have demonstrated that the functional characteristics of the bovine oviduct and uterus change with the age-dependent upregulation of inflammation and noted that S100A9 triggers inflammatory responses in oviduct epithelial cells. In the present study, we investigated the hypothesis that S100A9 affects reproductive events to aspect such as sperm function, fertilization, and the development of the embryo in cows. To investigate the effect of S100A9 on bovine sperm, we incubated sperms *in vitro* with S100A9 for 5 h and observed significantly decreased sperm motility and viability. During *in vitro* fertilization, S100A9 treatment for 5 h did not affect the rate of fertilization, time of first division of embryos, or embryo development to blastocyst stage. Treatment of 2-cell stage embryos with S100A9 for 5 h significantly reduced the proportion of cells undergoing normal division (4–8 cell embryos) and embryo development to the blastocyst stage. In experiment involving 24 h treatment of 2-cell embryos, the development of all embryos stopped at the 2-cell stage in the S100A9-treated group. In blastocyst-stage embryos, S100A9 treatment significantly stimulated the expression of endoplasmic reticulum (ER) and the mRNA expression of ER stress markers, and activated caspase-3 with subsequent nuclear fragmentation. Pre-treatment with an ER stress inhibitor significantly suppressed caspase-3 activation by the S100A9 treatment, suggesting that S100A9 induces blastocyst dysfunction by apoptosis (via caspase-3 activation) depending on ER stress. These results indicate that direct exposure to S100A9 exerted adverse effects on sperm function and embryo development. These findings suggest that excessive dose of S100A9 may have an adverse effect to the reproductive machinery by inducing inflammation and tissue dysfunction.

## Introduction

The reproductive events necessary to establish pregnancy are complex and well-controlled. The oviduct is an essential component of the female reproductive system and is important for oocyte maturation, sperm capacitation, fertilization, and the regulation of early embryo development, thereby determining the success of pregnancy [1]. Recently, there has been a decline in the fertility of lactating dairy cows in many countries. The fertilization rate of cattle is as high as 90% when appropriate artificial insemination is used, but the pregnancy success rate is below 50%, indicating that the rate of early embryo loss in cows is very high [2]. It has been suggested that the oviduct is crucial for creating an appropriate microenvironment for fertilization and early embryogenesis; therefore, dysfunction in the oviduct may lead directly to infertility [3].

Aging is the result of complex biological and environmental interactions, causing cellular and organismal dysfunction, and plays a critical role in fertility. It is well understood that the rate of pregnancy decreases significantly with the deterioration of oocytes due to aging [4, 5]. With aging, oocytes exhibit abnormal chromosome division, decreased mitochondrial quality, including the accumulation of mutations in the mitochondrial DNA and low ATP production, increased oxidative stress, and decreased levels of antioxidants [5–7]. We previously demonstrated that the functional characteristics of the bovine oviduct and uterus change with age-dependent upregulation of inflammation [3, 8]. We also reported that aged bovine oviduct epithelial cells expresses inflammation-related factors at high levels, especially S100A9, and S100A9 triggers of inflammatory responses in oviduct epithelial cells [9]. Additionally, S100A9 proteins exist in epithelial and stromal cells in bovine uterus [10]. Treatment with inflectional signal such as lipopolysaccharide (LPS) and Bacillus pumilus stimulates mRNA expression of S100A9 in bovine endometrium cells [10, 11]. Therefore, we hypothesized that S100A9 in bovine oviduct and uterus is associated with the initiation of inflammation and decline of reproductive function.

S100A9 is also known as calgranulin B and is constitutively expressed in immune cells, including neutrophils, monocytes, and dendritic cells. Swindell et al. [12] reported that shifts in the abundance of S100A9 are robust features of normal aging in mice. S100A9 can activate inflammatory responses and regulate apoptosis in various types of cells [13–15]. In addition, S100A9 are elevated during pregnancy-related complications such as preeclampsia, pregnancy loss, and recurrent early pregnancy loss in women [16, 17]. On the other hand, ovulatory stimulation by human chorionic gonadotropin induces S100A9 expression in cumulus-oocyte complexes, suggesting a physiological role for S100A9 in ovulation [18]. Although S100A9 plays a role in the induction of inflammatory cytokine production in oviduct epithelial cells [9], there are no investigation about the effect of S100A9 on germ cell function and embryonic development.

In the study reported here, we investigated whether S100A9 affects reproductive events such as sperm function, fertilization, and the development of embryos. The objectives of the present study were as follows: (1) to investigate the cytotoxic effects of S100A9 on sperm; (2) to determine the effects of S100A9 during *in vitro* fertilization (IVF) and subsequent embryo development; and (3) to assess any adverse effects of S100A9 on blastocyst-stage embryos using bovine sperm, oocytes, and embryos *in vitro*.

## Materials and methods

The protocol was approved by the local ethics committee of Tokyo University of Agriculture (No.300099).

### Treatment concentration of S100A9

In the present study, S100A9 (Cloud-Clone Corp Inc., Katy, TX) were purchased and used in vitro experiment. S100A9 was reconstituted by ultrapure distilled water (Thermo Fisher Scientific Inc., Waltham, MA). Previously, we reported that 0.1 and 0.5 μg/ml of S100A9 induced inflammatory cytokine secretion in bovine oviduct epithelial cells [19]. In bovine and human mononuclear cells, treatment of S100A9 (5–10 μg/ml each) upregulated inflammatory cytokine secretion [20, 21]). In our preliminary investigation for the present study, treatment with S100A9 at 0.1 and 0.5 μg/ml did not affect embryo development. Therefore, we selected high dose of S100A9 at 2 μg/ml or higher in the present study.

### Experiment 1: Effects of S100A9 on sperm function

#### Sperm preparation and in vitro culture of sperm

Frozen-thawed semen from a Japanese Black bull (three bulls, Livestock Improvement Association of Japan Inc., Tokyo, Japan) was used to investigate sperm function. The thawed semen was washed twice (at 700 g for 5 min) and sperm concentration was calculated.

To investigate the effect of S100A9 on sperm viability, semen at a concentration of 2 × 10^4^ cells/well, 100 μl/well, was seeded in a 96-well culture plate (Thermo Fisher Scientific Inc.) and treated with S100A9 (4 and 8 μg/ml) or same volume of distilled water as the control for 5 h at 38.5 °C in a humidified atmosphere with 5% CO_2_. In the present study, IVF was performed for 5 h due to suppress polyspermy fertilization, so the same time was set for sperm exposure of S100A9. Sperm viability was assessed using a LIVE/DEAD Sperm Viability Kit (Molecular Probes, Inc., Eugene, Oregon, USA) in accordance with the manufacturer’s protocol. Living sperm were showed bright green fluorescence, whereas dead and dying sperm produced red and yellow fluorescence, respectively. The stained sections were analyzed using a microscope (DMI6000, Leica Microsystems GmbH, Wetzlar, Germany) and about 1000 sperm were counted in each group (600X magnification).

To investigate the effect of S100A9 on ATP production, semen at 3 × 10^5^ cells/well, 100 μl/well, was seeded in a 96-well culture plate and treated with S100A9 (4 and 8 μg/ml) for 5 h. Sperm cell lysates were prepared using RIPA buffer (Wako Pure Chemical Industries, Osaka, Japan) with an ultrasonic crushing process. The ATP content was determined by measuring the luminescence generated using an ATP-dependent luciferin-luciferase bioluminescence assay (ATP assay kit; Toyo-Inc., Tokyo, Japan).

To investigate the effect of S100A9 on sperm motility, sperm motility parameters were analyzed using a computer-assisted semen analysis (CASA) machine (Ceros2, Hamilton Thorne Research Inc., Beverly, USA). Sperm samples (1 ml at 10 × 10^8^ cells) were divided into three groups: control, 4 μg/ml, and 8 μg/ml, and incubated for 5 h at 38.5 °C. Thirty frames from each sample were acquired at a frame rate of 60 Hz.

### Experiment 2: Effects of S100A9 on fertilization and subsequent embryonic development

#### IVF and subsequent embryonic development

*In vitro* maturation and fertilization were carried out as previously described [22–25]. In brief, bovine ovaries obtained from a local slaughterhouse (Central Wholesale Market, Shibaura, Tokyo, Japan) were transported to the laboratory. The cumulus-oocyte complexes (COCs) were aspirated from the follicles (2–5 mm in diameter) and washed three times in TCM-199 (Gibco BRL, Rockville, MD, USA) containing 20 mM HEPES supplemented with 5% fetal bovine serum (FBS; HyClone, GE Healthcare UK Ltd., Buckinghamshire, England). During the first wash, COCs surrounded by five or more layers of compact cumulus cells were selected for further experiment due to difference of performance depending on the layer of COCs [26]. The COCs were matured for 20–21 h at 38.5 °C in a humidified atmosphere with 5% CO_2_. Matured oocytes were inseminated with frozen-thawed semen from a single Japanese Black bull (adjusted to 2 × 10^6^ cells/ml) in 1 ml droplets for 5 h at 38.5 °C in a humidified atmosphere with 5% CO_2_ in the air, in 1 ml of BO solution containing 10 mg/ml bovine serum albumin and 10 μg/ml heparin.

To investigate the effect of S100A9 on fertilization, oocytes to sperm-oocytes were co-incubated with S100A9 (2 and 4 μg/ml) for 5 h during IVF. The day on which the IVF was conducted was designated as Day 0. Five hours later, fertilized oocytes at the pronuclear stage were washed and cultured in fresh synthetic oviductal fluid (SOF) medium as previously described [22, 23]. At Days 6, 7, and 8, the degree of embryonic development was checked and recorded.

### Experiment 3: Effects of S100A9 on 2-cell stage embryos derived from normal IVF

#### In vitro culture of embryos after IVF and subsequent embryonic development

To investigate the post-fertilization effects of S100A9 on the development of embryos derived by IVF, 2-cell stage embryos (Day 1) were treated with S100A9 (2 and 4 μg/ml) or same volume of distilled water as the control for 5 h, 24 h, or seven days (until Day 8). At Days 6, 7, and 8, the degree of embryonic development was checked and recorded.

### Experiment 4: Effects of S100A9 on ER stress of blastocyst

#### In vitro culture of blastocyst embryos after IVF

To investigate the effect of S100A9 on blastocyst-stage embryos, IVF was carried out and fertilized embryos were cultured to blastocyst-stage. These blastocysts were treated with S100A9 (4 μg/ml) or same volume of distilled water as the control for 24 h to examine active caspase-3 expression and endoplasmic reticulum (ER) stress. To further explore the relationships between S100A9 and ER stress, 4-phenylbutyric acid as an ER stress inhibitor (4-PBA, 50 μM, Wako Pure Chemical Industries) was applied before treatment with S100A9 in blastocyst embryo culture medium.

#### Detection of endoplasmic reticulum

To detect ER stress, ER tracker (Molecular Probes, Eugene, OR) was used according to the manufacturer’s instructions. Although ER tracker can selectively detect the membrane of the ER, the staining intensity of the ER tracker increases when ER stress is induced pharmacologically [27]. After treatment with or without S100A9 for 5 h, blastocyst embryos were treated with ER tracker reagents for 30 min at a concentration of 1 μM, washed with PBS three times, fixed with 3.7% paraformaldehyde for 15 min, and again washed with PBS three times. The cells to blastocysts were covered with VECTERSHIELD with 4′6-diamidino-2-phenylindole (DAPI) (Vector Laboratories, Inc. Burlingame, CA). The stained sections were analyzed using a microscope (Leica Microsystems GmbH, 400X magnification). The areas positive for fluorescence staining areas by ER tracker were extracted using Photoshop CC2018 software to calculate the area of the fluorescence as a percentage of the area of the image. Quantification was performed on 8 embryos from the control group and 10 embryos from the S100A9-treated group.

#### Measurement of caspase-3 activity

To detect caspase-3 activity, blastocyst embryos were stimulated with or without S100A9 (4 μg/ml) for 24 h. Caspase-3 activity was analyzed using the carboxyfluorescein FLICA caspase-3 assay kit (Immunochemistry Technologies, Bloomington, MN) according to the manufacturer’s instructions. The cells were covered with VECTERSHIELD with DAPI. The staining sections were analyzed using a microscope (Leica Microsystems GmbH, 400X magnification). The areas positive for fluorescence indicating active caspase-3, were identified using Photoshop CC2018 software, and the proportion of the image showing fluorescence was calculated. Quantification was performed on 6 embryos from the control group, 8 embryos from the S100A9-treated group, and 5 embryos from the S100A9 with 4-PBA group.

#### RNA extraction, cDNA production, and real-time PCR

Blastocyst embryos were stimulated with or without S100A9 (4 μg/ml) for 5 h. After incubation, embryos were collected using lysis buffer of RNAqueous Total RNA Isolation Kit (Thermo Fisher Scientific Inc.) to analyze mRNA expression, and stored -80 °C until analysis. Total RNA was prepared according to the manufacturer’s instructions. The cDNA production were performed with a commercial kit (ReverTra Ace; Toyobo Co., Ltd., Osaka, Japan). Real-time quantitative PCR was performed with the CFX Connect^TM^ Real Time PCR system (Bio-Rad, Hercules, CA, USA) and a commercial kit (Thunderbird SYBR qPCR Mix; Toyobo Co., Ltd.) to detect the mRNA expressions of *glucose-regulated protein 78 (GRP78)*, *activating transcription factor 4 (ATF4)*, *C/EBP homologous protein (CHOP)*, *beta-actin* (*ACTB*), *glyceraldehyde-3-phosphate dehydrogenase (GAPDH)*, *succinate dehydrogenase complex (SDHA)*, *or tyrosin 3-monooxygenase/tryptophan 5-monooxygenase activation protein zeta (YWHAZ)*. The primers used for real-time PCR were as follows: forward, 5′- GCATCGACCTGGGTACCACCTA -3′ and reverse, 5′- CCCTTCAGGAGTGAAAGCCACA -3′ for GRP78 (accession no. BC119953); forward, 5′- CTGGAGAGAAGATGGTAGCAGCAA -3′ and reverse, 5′- GCCCTCTTCTTCTGGCGGTA-3′ for ATF4 (accession no. BC151812); forward, 5′- GAACCTGAGGAGAGAGTGTTCCA -3′ and reverse, 5′- 5812AGTGACTCAGCTGCCATCTCTGT -3′ for CHOP (accession no. NM_001078163); forward, 5′- CAGAAGGACTCGTACGTGGG -3′ and reverse, 5′- TTGGCCTTAGGGTTCAGGG -3′ for ACTB (accession no. NM_173979); forward, 5′- ACAGTCAAGGCAGAGAACGG -3′ and reverse, 5′- CCACATACTCAGCACCAGCA -3′ for GAPDH (accession no. NM_001034034); forward, 5′- GCAGAACCTGATGCTTTGTG -3′ and reverse, 5′- CGTAGGAGAGCGTGTGCTT -3′ for SDHA (accession no. NM_174178); forward, 5′- GCATCCCACAGACTATTTCC -3′ and reverse, 5′- GCAAAGACAATGACAGACCA -3′ for YWHAZ (accession no. NM_174814). RT-qPCR was performed in duplicate with a final reaction volume of 20 μl containing 10 μl of SYBR Green, 7.8 μl of distilled water, 0.1 μl of 100 μM forward and reverse primers, and 1 μl of cDNA template. The amplification program consisted of a 5 min denaturation at 95°C followed by 40 cycles of amplification (95°C for 15 sec, 60°C for 20 sec, and 72°C for 10 sec). As internal control for analysis, ACTB, GAPDH, SDHA or YWHAZ were used and we confirmed that the results were similar regardless of which primer was used. The expression levels of each target gene were normalized to the corresponding ACTB threshold cycle (CT) values using the ΔΔ CT comparative method [28]. The relative amount of each PCR product was also calculated in comparison, using ACTB as the international standard. Quantification was performed on 8 embryos from the control group and 10 embryos from the S100A9-treated group.

#### Statistical analysis

All data are presented as means ± SEM. Differences between treatment groups were identified nonparametric analysis of variance, followed by Mann-Whitney U-test or Kruskal test. Probabilities less than 5% (*P* < 0.05) were considered significant.

## Results

### Experiment 1: Cytotoxic effects of S100A9 on sperm function

**Fig 1:**
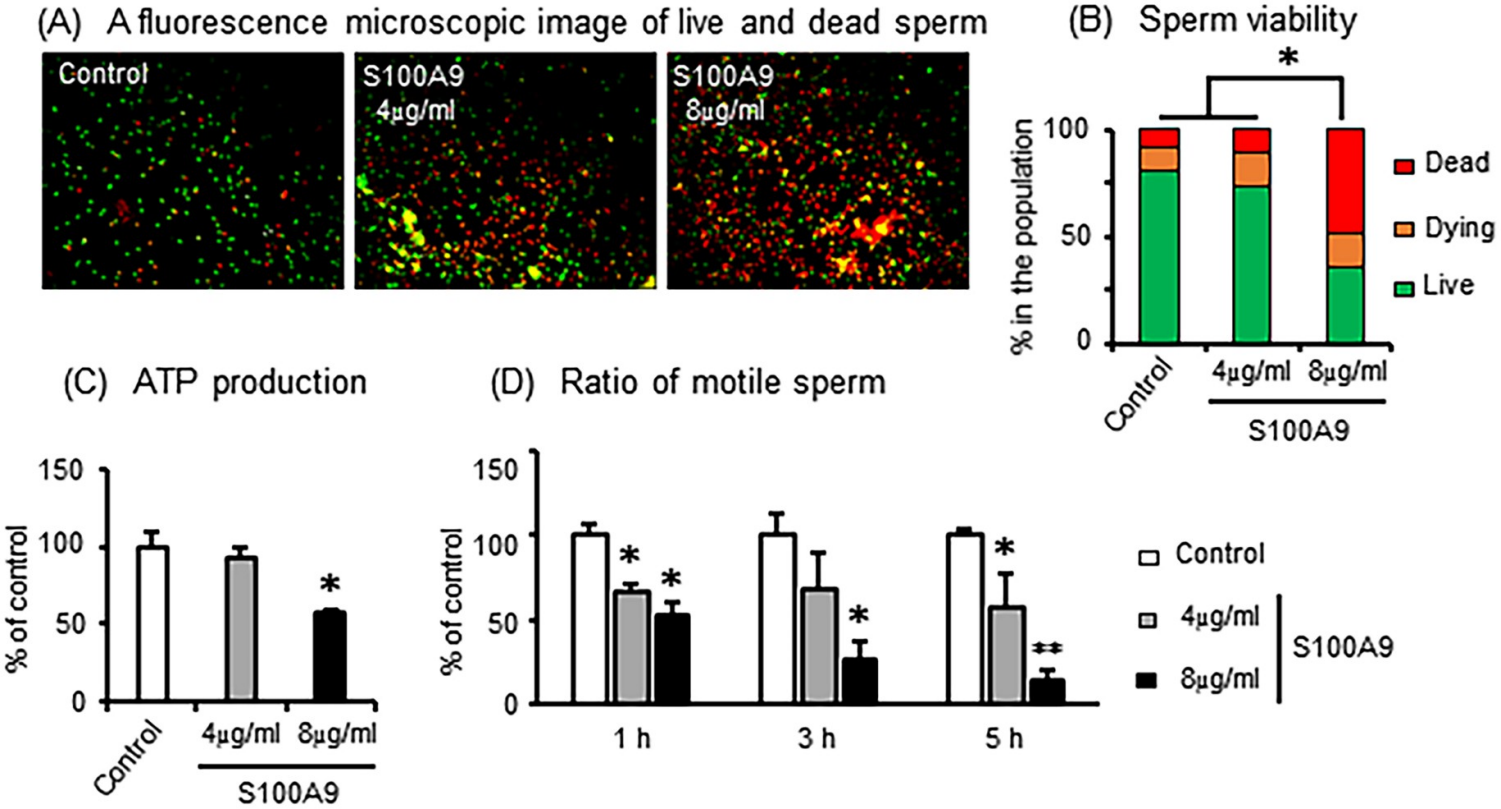
Effects of S100A9 on sperm viability and function. Bovine sperm was treated with 0, 4, and 8 μg/ml of S100A9 for 5 h. (A) Fluorescence microscopic image of live (green), dying (yellow), and dead (red) sperm. (B) Numbers of sperm in each condition were counted, and the population ratio was calculated. (C) ATP production within sperm was determined after 5 h of treatment with S100A9. (D) Sperm motility was observed using CASA and the ratio of motile sperm was calculated. All values are shown as mean ± SEM. ‘* or **’ indicates significant differences (*P* < 0.05 or 0.01).

To determine the cytotoxic effects of S100A9 on bovine sperm, sperms samples were treated with S100A9 at doses of 0 (control), 4, or 8 μg/ml for 5 h. Fluorescence microscopic images showed living (green stained by SYBR-14), moribund (yellow stained by SYBR-14 and propidium iodide), and dead (red stained by propidium iodide) sperms (Fig 1A). The number of each type of sperm was calculated by counting the proportion of green, yellow, and red fluorescence, respectively (Fig 1B). The proportion of dead sperm was significantly increased, and the proportion of living sperm dramatically decreased by high dose of S100A9 treatment (8 μg/ml) compared with the control group. A similar pattern was observed with respect to sperm viability (Fig 1B), the production of ATP by sperm was significantly inhibited by high dose S100A9 treatment (8 μg/ml) compared with that of the control group (Fig 1C). Using CASA analysis, we were able to quantitate changes in sperm kinematic parameters in response to exogenous factors. As shown in Fig 1D, both doses of S100A9 significantly reduced in the proportion of motile sperm in a dose- and time-dependent manner (Fig 1D). These findings suggest that high dose S100A9 treatment (8 μg/ml) induces sperm death, whereas the application of S100A9 at 4 μg/ml reduces sperm function by inhibiting motility.

### Experiment 2: Effects of S100A9 exposure on fertilization and subsequent embryonic development

To examine the effects of S100A9 during fertilization, sperm samples were treated with S100A9 for 5 h during IVF. Because high doses (8 μg/ml) of S100A9 made sperm unviable (Fig 1), lower concentrations (2 and 4 μg/ml) were used in the IVF experiment. After 5 h of IVF with or without S100A9, oocytes were washed and further cultured in flesh medium (without S100A9) until the next day. Two-cell stage embryos were judged have undergone normal divisions. As shown in Table 1, there were no adverse effects of S100A9 on fertilization, first division of embryos, or embryo development to blastocyst stage. These results indicated that on the S100A9 concentrations tested in this study, no impairment on fertilization and subsequent embryonic development was observed when the gametes were exposure during IVF.

**Table 1:** Effect of S100A9 on bovine sperm fertilization capacity in the IVF procedure.

Category	No. of oocytes (replicates)#	%. of division of embryos	% of embryo development (more than blastocyst stage)		
			Day 6	Day 7	Day 8
**Control**	200 (3)	73.0 ±3.4	17.5 ±3.8	13.5 ±3.1	39.7 ±5.3
**S100A9 2 μg/ml**	199 (3)	72.6 ±4.6	18.1 ±3.1	13.1 ±1.1	39.5 ±3.1
**S100A9 4 μg/ml**	216 (3)	72.6 ±5.7	16.7 ±2.1	14.4 ±0.9	42.8 ±1.2

^#^: 60–75 oocytes were used in each group in an experiment and the experiment were repeated 3 times.

### Experiment 3: Effects of S100A9 exposure on the development of 2-cell stage embryos derived from normal IVF

To examine the effects of S100A9 on embryo development, 2-cell stage embryos (Day 2) derived by normal IVF were incubated with or without S100A9 (2 and 4 μg/ml) for 7 days. As shown in Table 2, the group treated with 2 μg/ml S100A9 experienced a significant decline in the proportion of blastocysts compared with the control group at Days 6–8. At blastocyst stage, the total cell number of embryos was 101.0 ± 11.3 in the control (n = 7) and 47.2 ± 9.9 in S100A9-treated group (n = 6), indicating that S100A9 significantly decreased the cell number of blastocyst embryos (*P* < 0.05). In addition, 4 μg/ml S100A9-treated group had a drastic impact on 2-cell stage embryos, resulting that all embryos stopped developing at the 2-cell stage.

**Table 2:** Effect of S100A9 on 2 cell stage fertilized embryos.

Category	No. of 2 cell embryos (replicates)#	% of embryo development (more than blastocyst stage)		
		Day 6	Day 7	Day 8
**Control**	70 (4)	47.1±9.4	70.0±11.2	72.8±10.9
**S100A9 2 μg/ml**	90 (4)	8.9±5.5 **	37.7±10.4 **	43.3±8.8 **
**S100A9 4 μg/ml**	80 (4)	0.0±0.0 **	0.0±0.0 **	0.0±0.0 **

**, p<0.01, comparisons were made between the control group and each treated group

^#^: 10–25 embryos were used in each group in an experiment and the experiment were repeated 4 times.

We then investigated the effect of 4 μg/ml S100A9 on 2-cell stage embryos over time (Table 3). After 24 h of treatment over Days 2 to 3, all embryos completely stopped developing, as seen in the 7 days treated-experiment described above. On the other hand, 5 h treatment with S100A9 significantly reduced the proportion of embryos undergoing normal division (to produce 4–8 cell embryos). About 48% of 2-cell embryos divided normally after S100A9 treatment for 5 h. Moreover, about 60% developed to blastocyst stage after S100A9 treatment for 5 h, but the proportion of development to blastocyst stage was significantly lower than that in the control group.

**Table 3:** Time-dependent effect of S100A9 on 2 cell stage fertilized embryos.

Category	Treatment period	No. of 2 cell embryos (replicates)#	%. of division of embryos	% of embryo development (more than blastocyst stage)		
				Day 6	Day 7	Day 8
**Control**	24 h	40 (3)	97.6	52.5±10.1	80.0±13.2	72.5±5.7
**S100A9 4 μg/ml**		30 (3)	0 **	0.0±0.0 **	0.0±0.0 **	0.0±0.0 **
**Control**	5 h	30 (3)	100.0	63.3±6.6	87.6±6.6	83.3±8.8
**S100A9 4** **μg/ml**		60 (3)	48.3 *	36.6±4.4 *	66.6±5.9 *	60.0±8.6 *

* or **, p<0.05 or p<0.01, comparisons were made between the control group and each treated group

^#^: 10–20 embryos were used in each group in an experiment and the experiment were repeated 3 times.

### Experiment 4: Effects of S100A9 exposure on blastocyst stage embryos derived from normal IVF

**Fig 2:**
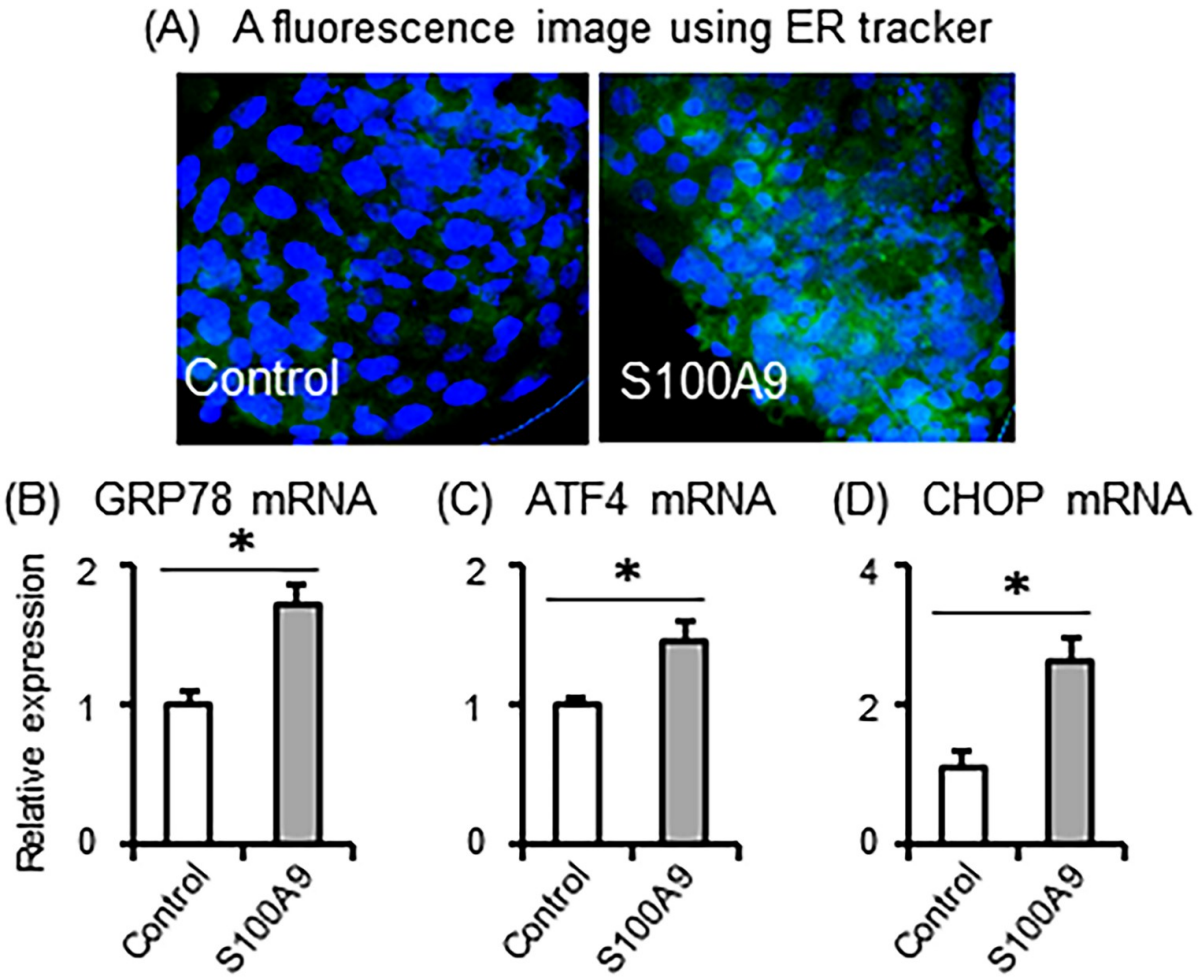
Effect of S100A9 exposure to blastocyst stage embryos on ER stress. Bovine blastocyst stage embryos were treated with 0 and 4 μg/ml of S100A9 concentrations for 5 h. (A) A fluorescence microscopic image of embryos by ER tracker. (B-D) The mRNA expression of GRP78, ATF4, and CHOP was determined (relative to ACTB mRNA levels). All values are shown as mean ± SEM. ‘*’ indicates significant differences (*P* < 0.05).

To examine the short-term effects of S100A9 on development of blastocyst, embryos from Days 7 to 8 derived from normal IVF were incubated with or without S100A9 (4 μg/ml) for 5 h. It is known that excessive ER stress leads to impaired embryonic development. As shown in Fig 2A, treatment with S100A9 clearly increased the intensity of ER tracker staining in treated embryos compared with control. In addition, S100A9 treatment significantly stimulated the mRNA expression of ER stress markers, including GRP78, ATF4, and CHOP, compared with the control (Fig 2B, 2C and 2D), indicating the induction of ER stress in blastocyst embryos by S100A9 exposure.

**Fig 3:**
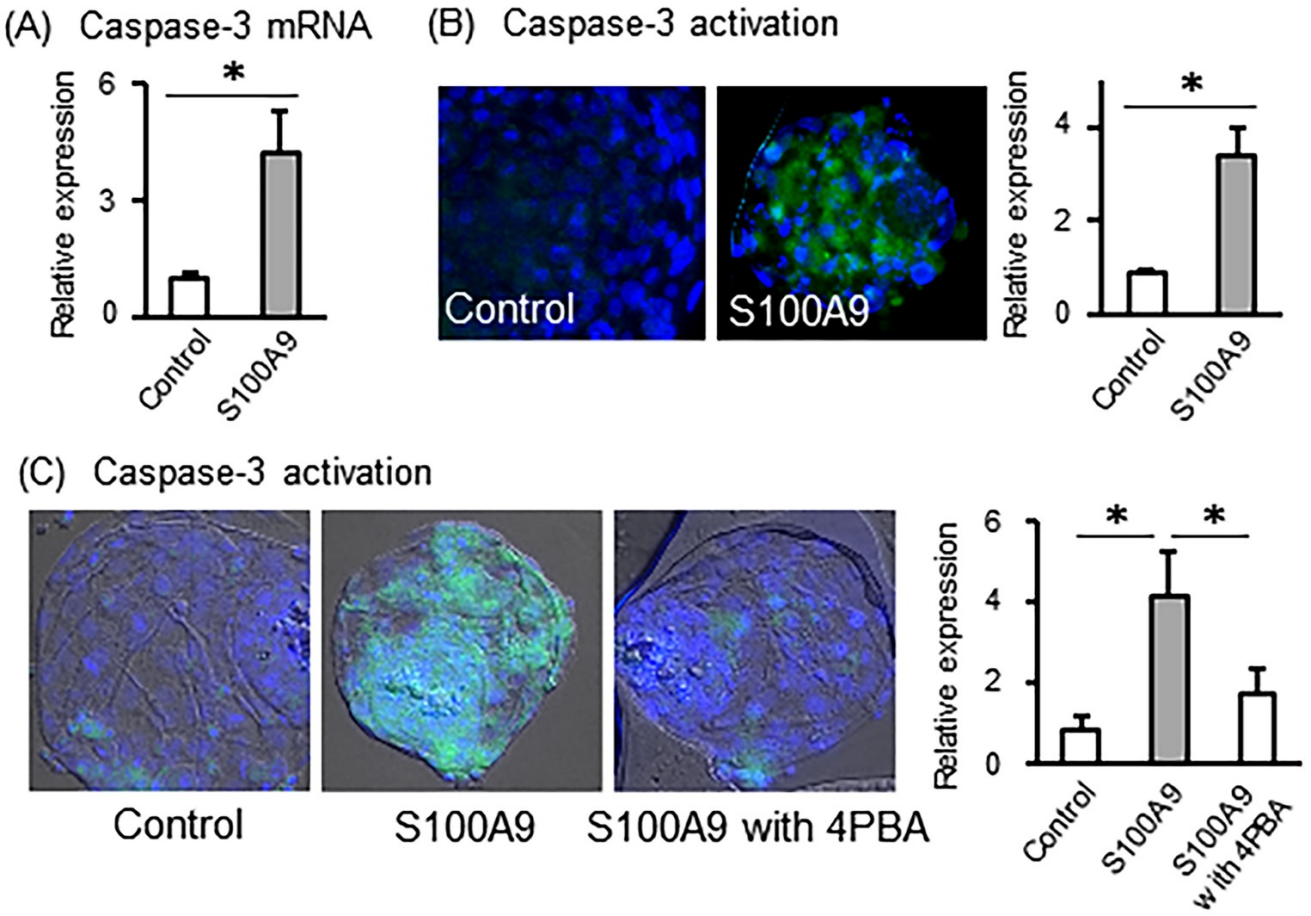
Effect of S100A9 exposure to blastocyst stage embryos on caspase-3 activation. Bovine blastocyst-stage embryos were treated with 0 and 4 μg/ml of S100A9 concentrations for 24 h. (A) The mRNA expression of caspase-3 was determined (relative to ACTB mRNA levels). (B) Fluorescence microscopic image of active caspase-3 in embryos and calculated the relative fluorescence expression levels. (C) Blastocyst embryos were pretreated with ER stress inhibitor then treated with S100A9. Fluorescence microscopic image of active caspase-3 in embryos and calculated the relative fluorescence expression levels. All values are shown as mean ± SEM. ‘*’ indicates significant differences (*P* < 0.05).

Finally, we investigated the long-term effects of S100A9 on developed embryos. Blastocyst stage embryos were incubated with or without S100A9 (4 μg/ml) for 24 h. S100A9 treatment significantly stimulated mRNA expression of caspase-3 compared with the control (Fig 3A). Furthermore, the treatment with S100A9 significantly increased caspase-3 activation in treated embryos compared with the control (Fig 3B). In addition, nuclear fragmentation was observed following S100A9 stimulation in blastocyst embryos. To examine the role of ER stress, blastocyst embryos were pre-treated with the ER stress inhibitor 4-PBA for 1 h, and then stimulated by S100A9. Pre-treatment with 4-PBA significantly inhibited activation of caspase-3 stimulated by S100A9 (Fig 3C).

## Discussion

As an alarmin of inflammation, levels of S100A9 are significantly increased in all types of inflammation, and S100A9 is associated with various types of inflammation-related diseases [14]. We showed previously that S100A9 is highly expressed in aged, as opposed to young, oviduct epithelial cells [9]. Thus, we hypothesized that excessive S100A9 from aging oviduct, not oocytes, decreases reproductive function, subsequently leading to infertility. In the present study, we investigated whether S100A9 affects reproductive events such as sperm function, fertilization, and early embryo development in vitro. We showed that the pathological dose of S100A9 suppressed sperm function, induced cell cycle arrest, and inhibited early embryonic development in cows.

Unsuitable microenvironments in reproductive tissues are a problem for early embryonic development and pregnancy. For example, treatment with LPS drastically stimulated mRNA expression of inflammatory cytokines in oviduct epithelial cells, and LPS challenge reduced the developmental rates of blastocyst stage embryos in co-cultures of embryos and oviduct epithelial cells [29]. Importantly, LPS treatment up-regulated expression of pro-inflammatory cytokines including S100A9 in bovine emdometrium [11]. Moreover, higher expression of S100A9 protein with infiltration of neutrophils was observed within the bovine uterus with a negative energy balance, this uterine environment were associated with poor reproductive performance [30]. These findings suggest that S100A9 adversely affects the reproductive machinery through both direct and indirect means by inducing inflammation of the oviduct and uterus.

Endoplasmic reticulum stress refers to a state in which cells are exposed various changes, and proteins are not normally folded in the lumen of the ER and accumulates as defective proteins. Induction of GRP78 is a well-known marker of ER stress. GRP78 is an essential regulator for ER stress due to its role as a major ER chaperone with anti-apoptotic properties as well as its ability to regulate for transmembrane ER stress sensors [31]. In mammalian cells, PERK, IRE1, and ATF6 exist as classical stress sensor proteins that sense ER stress. PERK signaling induces translation of ATF4, which translocates to the cell nucleus to regulate gene expression including CHOP [32]. CHOP is a transcription factor that regulates apoptosis in response ER stress. ER-stress induce apoptosis mediated through CHOP in various diseases, indicating the CHOP is major marker and execution factor of ER-stress. Data in the present study indicate that treatment with S100A9 induced the increase of ER stress markers (GRP78, ATF4, and CHOP) and caspase-3 activation in blastocyst-stage embryos, suggesting the occurrence of ER stress and apoptosis. In addition, pre-treatment with ER stress inhibitor 4-PBA in conjunction with S100A9 suppressed the activation of caspase-3 in blastocysts, indicating that S100A9 stimulates caspase-3 activation through ER stress. Indeed, Basar et al. [33] reported that ER stress inducer (tunicamycin) drastically reduced mouse blastocyst formation from 79% in control to 4% in treated individuals, with induction of nuclear fragmentation, and the ER stress inhibitor (tauroursodeoxycholic acid) reduced the adverse effects of the ER stress inducer. Moreover, the treatment with an ER stress inhibitor in mouse embryo culture medium attenuated ER stress-induced apoptosis and improved the implantation and livebirth rates of transferred mouse embryos [34]. These findings suggest that suppressing the increase in ER stress may lead to an improvement in embryo development rate and pregnancy rates. However, although the number of embryos analyzed in the present study was very small, it will be necessary to examine the effect of S100A9 on ER stress in future study.

The data show that treatment with S100A9 over 5 h during IVF did not affect the developmental rate to blastocyst-stage embryo, whereas 7 days treatment with S100A9 drastically induced cell cycle arrest at 2-cell stage embryos and inhibited the developmental rate to blastocyst embryos. We hypothesized that differences in the duration of S100A9 treatment result in different effects on early embryonic development. This hypothesis was partially confirmed by the results that S100A9 had adverse effects on early embryonic development in time-dependent manner as shown in Table 3. However, the reason why a decrease in sperm motility is not reflect following S100A9 addition in IVF, despite the same length of S100A9 treatment, is not clear. Although we conducted IVF for 5 h in the present study, it is known that sperm has already entered cumulus cells within 15 min of initiation of IVF [35]. In addition, Bungum et al. [36] have demonstrated that an incubation time as short as 30 sec is enough to obtain good fertilization and embryo development rates in human IVF. Therefore, the sperm that approached oocytes immediately after the onset of IVF was less affected by S100A9 exposure, so it is possible that no major damage was observed in subsequent embryonic development. However, in fact *in vivo*, since spermatozoa are present in the oviduct for several hours due to capacitation, if high concentration of S100A9 is produced from the oviduct, there is a possibility that the sperm may also be adversely affected, resulting the reduction of fertilization and embryo development. In future study, as with other reproductive toxicological studies [37–39], it will be necessary to perform IVF using spermatozoa to which have been previously exposed to S100A9 in order to determine its influence on embryonic development.

Normal levels of S100A9 in plasma are about 0.2–0.5 μg/ml in adult humans, and S100A9 levels in umbilical cord blood are very high, at 3 μg/ml even in healthy normal pregnancies [40]. On the other hand, there are no published data about the concentration of S100A9 in cows. In preliminary our data, the plasma concentration of S100A9 are about 0.1–0.5 μg/ml during the estrous cycle in Japanese black cattle. In inflammation-related diseases, the concentration of S100A9 in serum reaches levels of 5–10 μg/ml or more [41]. These data expected that S100A9 concentrations are different greatly depending on the blood collection site and the individual condition. Unfortunately, we did not measure S100A9 concentration in oviduct fluid and supernatant of oviduct epithelial cells (both from young and aged cows) because of below the detection limit. In the present study, the effective dose of S100A9 are 2–8 μg/ml and the physiological levels of S100A9 (0.1–1 μg/ml) did not affect embryonic development in our preliminary experiment. Thus, it is unlikely that the dose of S100A9 used in the present study will affect sperm or oocyte in physiological or healthy conditions. Therefore, the present data that S100A9 has a reproductive toxicity effect is considered to represent the state of acute inflammation or pathological conditions such as sever diseases rather than the verification of chronic inflammation of S100A9 with aging.

Although many experiments using S100A9 in cell culture have been reported, the concentrations used differ considerably depending on the cells used. For example, in bovine mononuclear cells, co-treatment of S100A9 and S100A8 (5 μg/ml each) upregulated interleukin-1β secretion [20]. In human peripheral blood mononuclear cells, S100A9 at a concentration of 10 μg/ml induced secretion of inflammatory cytokines [21]. In human chondrocytes, 1 μg/ml S100A9 stimulated inflammatory cytokine secretion, whereas in bovine oviduct epithelial cells S100A9 at lower concentrations (0.1 and 0.5 μg/ml) clearly induced inflammatory cytokine secretion [19]. Therefore, sensitivity to S100A9 may differ depending on cell type.

## Conclusions

Taken together, the results of the present study indicate that direct exposure to excessive dose of S100A9 exerted adverse effects on sperm function and embryo development. In particular, S100A9 reduced sperm viability and motility, suppressed early embryonic development, and induced blastocyst dysfunction by apoptosis (caspase-3 activation) depending on ER stress.
